# Experiences of Justice-Involved People Transitioning to HIV Care in the Community After Prison Release in Lusaka, Zambia: A Qualitative Study

**DOI:** 10.9745/GHSP-D-22-00444

**Published:** 2023-04-28

**Authors:** Helene J. Smith, Michael E. Herce, Chilambwe Mwila, Peter Chisenga, Chisenga Yenga, Besa Chibwe, Vivien Mai, Lillian Kashela, Mirriam Nanyagwe, Sisa Hatwiinda, Clement N. Moonga, Maurice Musheke, Yotam Lungu, Izukanji Sikazwe, Stephanie M. Topp

**Affiliations:** a Centre for Infectious Disease Research in Zambia, Lusaka, Zambia.; b School of Population Health, University of New South Wales, Sydney, Australia.; c Institute for Global Health and Infectious Diseases, University of North Carolina, Chapel Hill, NC, USA.; d Dalla Lana School of Public Health, University of Toronto, Toronto, Canada.; e Zambia Correctional Service, Government of the Republic of Zambia, Lusaka, Zambia.; f College of Public Health, Medical and Veterinary Sciences, James Cook University, Townsville, Australia.

## Abstract

**Introduction:**

In sub-Saharan Africa (SSA), incarcerated people experience a higher HIV burden than the general population. While access to HIV care and treatment for incarcerated people living with HIV (PLHIV) in SSA has improved in some cases, little is known about their transition to and post-release experience with care in the community. To address this gap, we conducted a qualitative study to describe factors that may influence post-release HIV care continuity in Zambia.

**Methods:**

In March–December 2018, we recruited study participants from a larger prospective cohort study following incarcerated and newly released PLHIV at 5 correctional facilities in 2 provinces in Zambia. We interviewed 50 participants immediately before release; 27 (54%) participated in a second interview approximately 6 months post-release. Demographic and psychosocial data were collected through a structured survey.

**Results:**

The pre-release setting was strongly influenced by the highly structured prison environment and assumptions about life post-release. Participants reported accessible HIV services, a destigmatizing environment, and strong informal social supports built through comradery among people facing the same trying detention conditions. Contrary to their pre-release expectations, during the immediate post-release period, participants struggled to negotiate the health system while dealing with unexpected stressors. Long-term engagement in HIV care was possible for participants with strong family support and a high level of self-efficacy.

**Conclusion:**

Our study highlights that recently released PLHIV in Zambia face acute challenges in meeting their basic subsistence needs, as well as social isolation, which can derail linkage to and retention in community HIV care. Releasees are unprepared to face these challenges due to a lack of community support services. To improve HIV care continuity in this population, new transitional care models are needed that develop client self-efficacy, facilitate health system navigation, and pragmatically address structural and psychosocial barriers like poverty, gender inequality, and substance use.

## Abstract (precis)

HIV care for incarcerated PLHIV in sub-Saharan Africa has improved, but little is known about their post-release experience with care. The authors conducted a qualitative study to describe factors influencing post-release HIV care continuity in Zambia.

## Teaser

**Key Findings:**

- Community health systems can be highly intimidating, overwhelming, and unaccommodating to people living with HIV (PLHIV) involved with the criminal justice system (i.e., the “justice-involved”) and particularly challenging to navigate after prison release.
- Justice-involved PLHIV in Zambia are highly marginalized and face a number of psychological, socioeconomic, and structural challenges, which they are typically ill-prepared and unsupported to manage after release. Without differentiated support tailored to their unique needs, these challenges may disrupt their HIV care continuity, resulting in HIV treatment interruption.

**Key Implication:**

- Dedicated and appropriately tailored interventions, which could include pre-release counseling and case management, post-release peer navigation, and/or psychosocial, socioeconomic, and gender-responsive supports, are needed to guide justice-involved PLHIV through the transition from accessing HIV care during incarceration to successfully accessing it in the community after release.

## BACKGROUND

Globally, more than 30 million people annually move between correctional facilities and other carceral settings and the community.^1^ In Zambia, a landlocked country in sub-Saharan Africa (SSA), nearly 23,000 people are incarcerated at any given time.^2^ HIV prevalence in this population is estimated to range from 14.3% to 27.4%,^3^^–^^7^ rates that are considerably higher than that seen in the general population.^8^ There is increasing recognition that to achieve the “95-95-95” goals set by the Joint United Nations Programme on HIV/AIDS for 2030,^9^ greater attention must be given to ensuring high levels of HIV testing, antiretroviral therapy (ART) initiation, and treatment maintenance among incarcerated and other key populations.^10^

While mounting evidence from SSA suggests that delivering HIV treatment and care is both feasible and highly effective within carceral settings,^4^^,^^6^^,^^11^^–^^15^ there is increasing concern that any individual and public health benefits gained during incarceration may be lost upon release unless adequate services are in place to link people to post-release HIV care in the community.^16^^–^^20^ Such a break in care continuity undermines the health of people who have a history of interaction with the criminal justice system (i.e., “justice-involved” people)^21^ and may perpetuate onward HIV transmission in the community.^16^ This problem merits special attention given the frequency with which incarcerated people move into, out of, and between carceral settings in SSA^3^^,^^22^^,^^23^ and data suggesting that justice-involved persons are more likely to engage in HIV risk behaviors before, during, and immediately after incarceration.^13^^,^^15^

A break in care continuity undermines the health of people who have a history of interaction with the criminal justice system and may perpetuate onward HIV transmission in the community.

While scarce research has examined this problem in SSA, studies from North America have explored factors contributing to interruptions in HIV care among releasees. Stigma related to both HIV status and incarceration history, coupled with competing hierarchies of basic needs like housing and food, have been identified as key factors in disrupting HIV care continuity in the United States.^24^^–^^26^ Other factors such as mental health, social stigma, and ongoing conflict with law enforcement^27^ have also been shown to limit HIV care access for releasees. Notably, substance use has been shown to be a key factor in disrupting HIV care post-release.^28^^,^^29^ Finally, a releasee’s past history of HIV care engagement is thought to be predictive of future retention in care.^30^ However, no comparable insights are available from countries in SSA.

To address this knowledge gap, we conducted a qualitative study with a sample of people living with HIV (PLHIV) who were newly released from prison in Zambia to describe factors that may influence post-release HIV care continuity.

## METHODS

### Study Design

This longitudinal qualitative study was part of a larger prospective cohort study, the Releasee Care Continuum (RCC) study (ClinicalTrials.gov: #NCT02905162). Between March and December of 2018, we recruited 50 participants from the RCC cohort to complete 2 in-depth interviews (IDIs). IDIs were conducted just before prison release and approximately 6 months after release to coincide with other RCC study procedures. Insights from the IDIs were supplemented by demographic and behavioral data obtained through questionnaires from the main RCC study.

### Study Setting and Description of Corrections HIV Program

Participants were recruited from 5 correctional facilities in Lusaka and Central Provinces, Zambia. These correctional facilities represented maximum security, midlevel, and open-air farm carceral settings typical for Zambia in that they all reported a high prevalence of overcrowding and HIV among incarcerated persons.^2^ HIV testing, treatment, and care were available from a clinic located within the men’s section of each facility and staffed by a mixture of Ministry of Health and Zambia Correctional Service health staff. Women, who generally reflect less than 5% of the incarcerated population in Zambia, were serviced by the same clinic located in the male section. In addition, prison health services were supported by a cadre of trained peer educators—incarcerated persons who work with clinic and corrections staff to provide health counseling, facilitate sputum sample collection for TB screening, and increase access to the clinic and HIV services for incarcerated persons. All clinics received external support to provide HIV care from nongovernmental organizations funded by various donors, including the U.S. President’s Emergency Plan for AIDS Relief.

Voluntary HIV testing services were offered as part of routine prison entry procedures at the study sites. If found to be HIV positive and wanting to initiate ART, incarcerated persons were enrolled in the national HIV treatment program with health information captured in the national electronic health record, SmartCare. Incarcerated persons who entered a correctional facility with a known HIV diagnosis were able to resume or continue ART following a confirmatory HIV test. Medication is dispensed monthly at the prison clinic but distributed daily, typically through directly observed therapy administered by each incarcerated person’s cell captain or chairman (a senior-ranking incarcerated person appointed within each cell with both disciplinary and welfare-related responsibilities). At release, standard procedures should involve providing individuals with brief discharge counseling and a referral letter to facilitate linkage to ART at a government clinic in the community where they can continue HIV treatment free of charge.

### Participants

We approached a purposive sample of RCC participants to join the qualitative component of the study beginning in July 2018. Participants were eligible for the qualitative study if they met inclusion criteria for the RCC study, including: being aged 18 years or older; being incarcerated at 1 of 5 prison study sites; having an anticipated release date within 30 days of study screening; having documented evidence of HIV infection and enrollment in the national HIV program; if on treatment, had been receiving ART for 30 days or more; planned to live in the Lusaka area upon release; were willing and able to provide locator information; and were willing and able to provide written informed consent in Nyanja, Bemba, or English. Purposive recruitment was done to ensure a balance of participant characteristics across the following variables: gender; sentenced versus awaiting trial; HIV care status (i.e., engaged in HIV care versus disengaged from HIV care); history of hazardous alcohol use at enrollment (yes vs. no); and major depressive or post-traumatic stress disorder symptoms at enrollment (yes vs. no).

### Materials

#### IDI Guide

IDI guides were informed by a conceptual model, the Behavioral Model for Vulnerable Populations,^31^ which offers an empirically supported framework to understand the combination of multilevel factors, termed “predisposing” (i.e., demographic and social structure characteristics), “enabling” (i.e., personal and family resources), and “need” (i.e., perceptions and evaluated needs of special relevance to vulnerable populations) factors, that may influence post-release HIV care retention and resulting HIV clinical outcomes. IDI guides explored these factors from our conceptual model, with a particular focus on: individual health beliefs; housing; food security; alcohol and drug use; internalized and enacted stigma; psychosocial support; experiences with, or perceptions of, HIV care in the community; and attitudes surrounding HIV status disclosure and adherence to ART and other medications. Additionally, the pre-release guide asked participants to describe their HIV care both before and during their incarceration, including their experiences with an HIV-positive diagnosis and how and when they came to be on ART, as well as their expectations around accessing HIV care once released. The post-release guide asked participants to pick up their story from when they were released to explore enabling and need factors. The IDI guides used at pre- and post-release are provided in the Supplement.

#### Supplemental Demographic and Psychosocial Questionnaire Data

To assist with purposive sampling and to better contextualize participant interview responses, we reviewed questionnaire data obtained from qualitative study participants in the main RCC study at both pre- and post-release time points. Pre-release questionnaires examined participant demographics while post-release questionnaires assessed for hazardous alcohol and drug use using the World Health Organization–validated Alcohol Use Disorder Identification Test^32^ and Drug Use Disorder Identification Test,^33^ respectively.

### Recruitment Procedures

Study staff approached RCC participants about the qualitative study in person and asked if they would like to participate. Study staff were already known to potential participants after having interacted with them previously during the main RCC study. After participants gave their written informed consent for the qualitative study, study staff arranged for an appropriate time to complete the pre-release IDI, which was audio-recorded. Pre-release IDIs were conducted in a private clinic room in the correctional facility without the presence of corrections staff. Each participant was free to stop the interview or withdraw at any time.

Post-release, once a participant had become eligible for follow-up in the main RCC study (approximately 6 months post-release), study staff commenced community follow-up activities. Once located and if renewed consent was provided, a post-release interview was scheduled and again audio-recorded. Participants were free to refuse a post-release follow-up interview or stop the interview or withdraw at any time.

### Data Collection and Analysis

Members of the study team transcribed and translated into English all interviews verbatim and uploaded translated transcripts into NVivo (QSR International) for coding. Two independent coders coded 5 transcripts each to refine and validate the codes followed by another 5 transcripts each to achieve consistency in coding. The remaining transcripts were then coded independently. Data were indexed by themes as well as demographic information, including gender, age, and HIV viral suppression status pre- and post-release. A thematic framework was developed for data analysis using both inductive reasoning based on emerging themes and deductive reasoning based on a priori themes from our conceptual model. The qualitative analysis applied a phenomenological perspective to the lived pre- and post-release experiences of participants to elucidate factors that enabled or impeded HIV care continuity.

### Case Classification

Each participant received a case classification to provide context when reviewing each transcript. Case classifications were allocated based on whether a participant’s HIV control changed from the pre-release to the post-release period according to the following viral load status categories: “undetectable” (defined as <20 copies/ml), “suppressed” (defined as 20–999 copies/ml), and “uncontrolled” (defined as ≥1,000 copies/ml). Participants were categorized according to the following case definitions: (1) unchanged undetectable/suppressed status; (2) unchanged uncontrolled status; (3) status improved (e.g., went from uncontrolled to suppressed/undetectable or from suppressed to undetectable); (4) status deteriorated (e.g., went from undetectable to suppressed or uncontrolled or from suppressed to uncontrolled); or (5) became lost to follow-up (LTFU). The final category identified the participant as LTFU to the study. It is possible that these participants did continue treatment; however, they may have moved out of the study area or continued treatment outside of the government health care system. The HIV control categories for participants are described in the Table and presented alongside any verbatim quotes.

**TABLE.** Demographic, Viral Load, and Alcohol Risk-Level Characteristics Among Justice-Involved People Living With HIV in Zambia

**Characteristics**	**No. (%)** **(N=50)**
Sex and age	
Male	40 (80)
Female	10 (20)
Age, median (range), years	34.1 (20–54)
Highest education level completed	
No education	1 (2)
Primary school	27 (54)
Secondary school	21 (42)
Higher than secondary school	1 (2)
Enrollment site	
Lusaka Central Correctional Facility	33 (66)
Kabwe Correctional Complex	14 (28)
Mwembeshi Correctional Facility	3 (6)
History of prior incarceration	7 (14)
Reason for release	
Sentence completed	46 (92)
Paroled	3 (6)
Released from court (i.e., remandee)	1 (2)
Viral load status change (from pre- to post-release)^a^	
Unchanged “undetectable” or “suppressed” (favorable) status	19 (38)
Unchanged “uncontrolled” (unfavorable) status	7 (14)
Viral load status improved	6 (12)
Viral load status deteriorated	11 (22)
Viral load status unknown (i.e., LTFU)	7 (14)
**Post-release alcohol use (for participants who completed a post-release survey, n=27)**	
Risk level for alcohol use disorder^b^	
Low risk	21 (78)
Hazardous or harmful alcohol use	6 (22)
Moderate-to-severe alcohol use	1 (4)

Abbreviations: AUD, alcohol use disorder; AUDIT, Alcohol Use Disorders Identification Test; LTFU, lost to follow-up; WHO, World Health Organization.

^a^ HIV viral load status categories include: undetectable (<20 copies/ml); suppressed (20–999 copies/ml); uncontrolled (≥1,000 copies/ml).

^b^ Risk level for AUD determined from the WHO AUDIT tool with the following scores (on scale from 0 to 40) for each category: low risk (1–7); hazardous or harmful (8–14); moderate-to-severe (≥15).

### Ethical Approval

The study was approved by the Zambian Ministry of Health, Lusaka Provincial Health Office, University of Zambia Biomedical Research Ethics Committee (#001-02-16), University of North Carolina at Chapel Hill (United States) Institutional Review Board (#16-0276), and James Cook University (Australia) Human Research Ethics Committee (#H6896).

## RESULTS

### Participant Flow and Demographic Information

**FIGURE 1:**
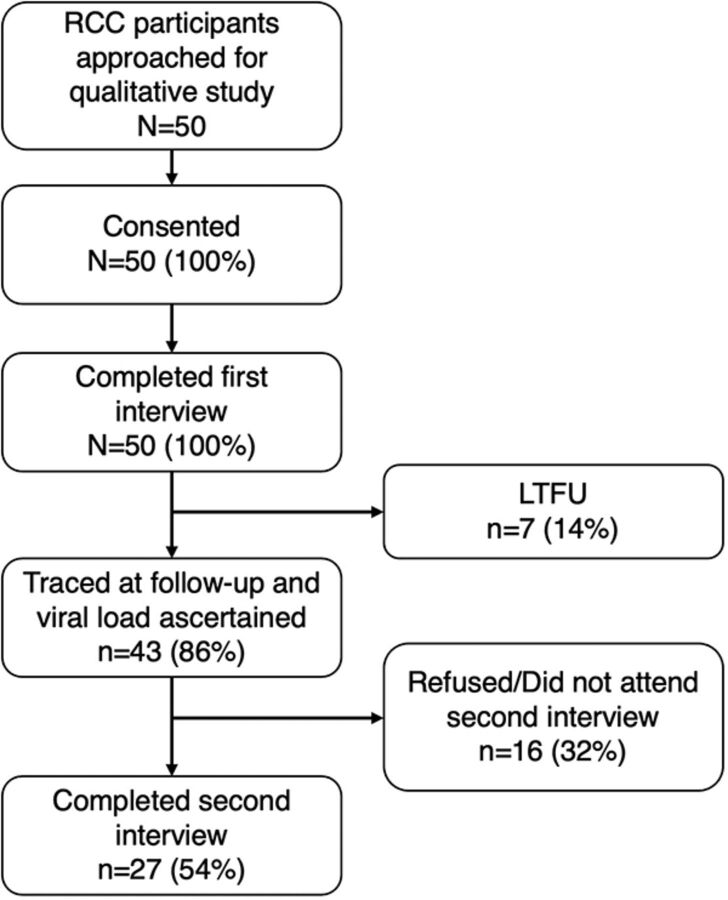
Enrollment Flow for Study of Factors Influencing Post-Release Care Among Justice-Involved People Living With HIV in Zambia Abbreviations: LTFU, lost to follow-up; RCC, releasee continuum cohort.

Fifty participants (N=50) consented, enrolled, and completed a pre-release IDI. At post-release follow-up, of the 50 who had completed pre-release interviews, 7 (14%) could not be traced and were LTFU; 16 (32%) either declined to participate in a post-release follow-up interview or, if an interview time was scheduled, failed to attend. This resulted in 27 participants completing both pre- and post-release interviews and 23 completing only 1 interview pre-release (Figure 1).

The Table shows the demographic, viral load, and alcohol risk-level characteristics of the participants. The majority of participants (40 [80%]) were male; however, compared to standard gender distributions within the Zambian correctional system (normally ∼3% women and ∼97% men), women were overrepresented at 10 participants (20%).^2^ Participants were aged 20–54 years (median age, 34.1 years). More than half (28 [56%]) never attended secondary school. The vast majority were incarcerated persons who had been sentenced (46 [92%]) and had not been previously incarcerated (43 [86%]).

Comparing the evaluation of viral load status at follow-up with status pre-release resulted in 19 (38%) of the participants classified as having an unchanged favorable status, with their viral load remaining “undetectable” or “suppressed”; 7 (14%) as having an unchanged unfavorable status, with their viral load remaining “uncontrolled”; 6 (12%) as status improved; and 11 (22%) as status deteriorated. Of the 27 participants who completed a post-release interview, 7 (26%) were at risk of hazardous or harmful alcohol use.

**FIGURE 2:**
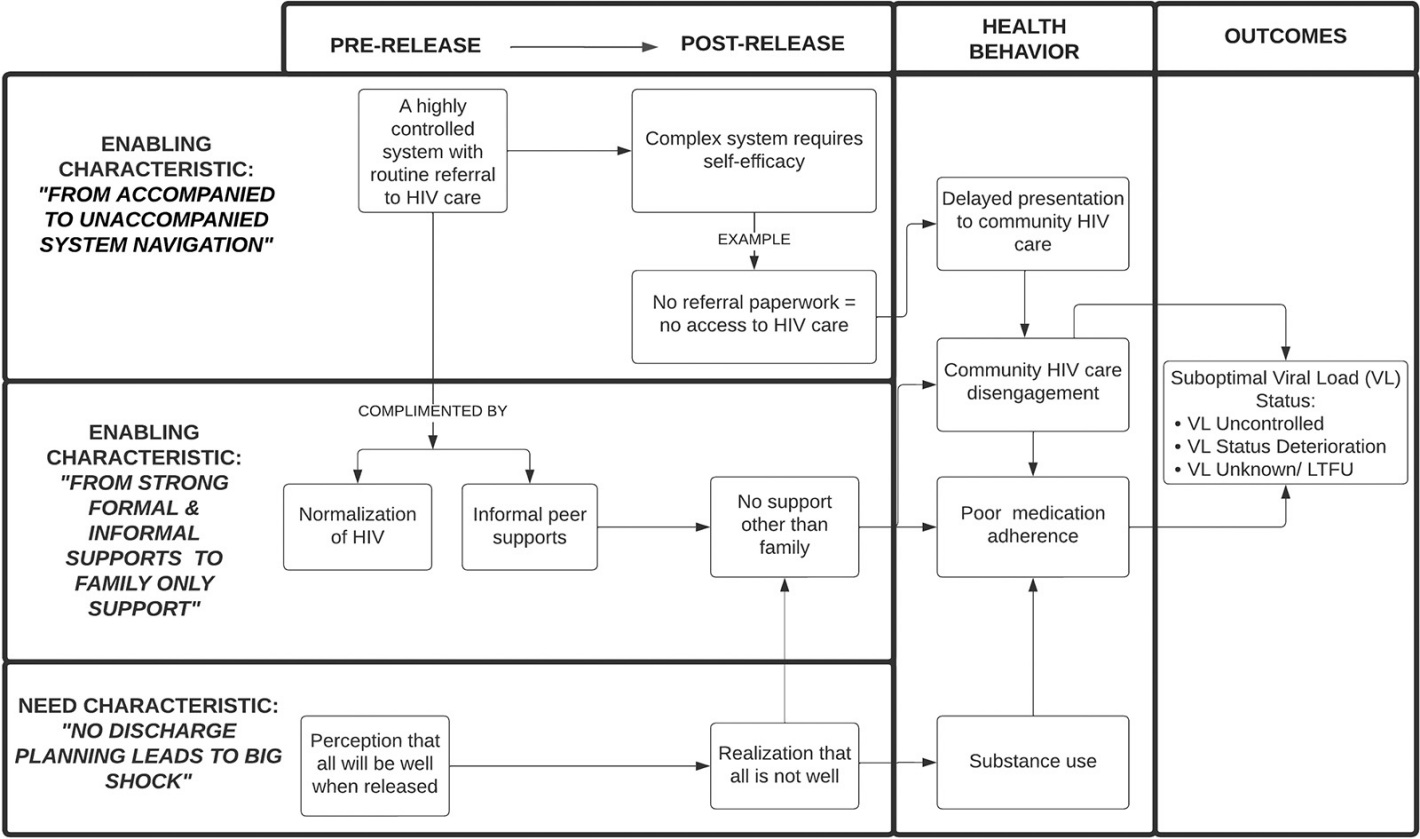
Thematic Map of Key Enabling and Need Characteristics Across the Pre- and Post-Release Periods for Justice-Involved People Living With HIV in Zambia Abbreviation: LTFU, lost to follow-up.

Using domains adapted from the Behavioral Model for Vulnerable Populations,^31^ we present key “enabling” and “need” characteristics as themes that either impeded or supported linkage to and retention in HIV care during both the pre-release (i.e., incarceration) and post-release periods. Figure 2 illustrates how these characteristics changed from the pre- to post-release periods.

### Enabling Characteristics: From Straightforward Access to Navigating a Complex System

#### Pre-Release: A Highly Controlled System With Routine Referral to HIV Care

Participants frequently attributed their ART adherence to the structured processes that promoted routine access to directly observed therapy within the corrections HIV program.

*But the chairman is strict and notices. He says, “come,” if he gives you the medication and if you say, “I will go and take it from my bed,” he will say, “no take it from here.” […] he has to be sure that you have taken your medication.* —Male, aged 39 years, became LTFU

Participants frequently attributed their pre-release ART adherence to the structured processes that promoted directly observed therapy within the corrections HIV program.

While most participants recognized the benefits of the structured system, a number also noted downsides. For example, the controlling nature of the prison system meant incarcerated individuals had little agency in situations where the system failed (e.g., where access to health care became limited by prison routine and procedures such as a lockdown) and/or in cases of abuse of power (e.g., cell captains arbitrarily choosing to withhold medication or duty officers gatekeeping access to the clinic).

*Sometimes when you are not feeling well, when you go [to the clinic] they will just say, “no, you were there just yesterday.” But only the sufferer understands the real extent of one’s pain, that’s the difference from being outside. I am free [on the outside].* —Male, aged 34 years, unchanged favorable status

#### Post-Release: Complex System Requires Self-Efficacy

At release, while most participants clearly expressed a desire to be free rather than incarcerated, they also recognized that they had lost some important structural and social supports that existed within prison. As a result, the onus to transition and remain engaged in HIV care outside corrections fell much more on the individual and their ability to problem-solve and self-advocate. Some participants were able to do this, coming up with creative solutions when unable to access the health care system due to a missing clinic referral letter, for example. A common workaround was to present to a clinic under the guise of being a new patient who did not know their status. These individuals had internalized their diagnosis, had a positive outlook on the future, and expressed feelings of self-love and self-confidence. For them, challenges associated with accessing the clinic were logistical in nature and could be overcome.

*Yes, I managed because with health you have to take care of yourself. If you love yourself, you need to do what your heart tells you to do. I can forget something else maybe to like eat, but I cannot forget to take the medication… Because I love myself.* —Male, aged 29 years, unchanged favorable status

For others, however, the same challenges were experienced as overwhelming and impossible to negotiate. The accounts of these participants often lacked the same self-affirming language as expressed in the quote above. For these participants, their attempts to sustain ART were derailed by poverty, mental health issues, both external and internal stigma related to their HIV status and incarceration history, and substance use, with little to no social or structural support to alleviate these issues.

*Once I have the alcohol in my blood, there is nothing else. I forget my troubles, that I am sick… and so you see it is easy to forget [to take my ART]… —*Female, aged 35 years, status deteriorated

#### Post-Release: No Referral Paperwork = No Access to HIV Care

For some participants, linking to HIV care in the community was a straightforward experience, involving the acquisition of a clinic referral letter or SmartCare card (containing encrypted health record information) from the releasing prison and attending a “prison-friendly” clinic. These clinics were community-based but located next to prisons and routinely provided services to incarcerated people. Consequently, health care staff at these facilities were often known to releasees, and participants felt comfortable disclosing their incarceration and clinical histories to staff. Importantly, these clinics were more accustomed to providing flexible, patient-centered health services.

However, navigating community health systems was not always straightforward and often hampered by highly bureaucratic requirements at the receiving clinic in the community. Not being in possession of a referral letter or SmartCare card made access particularly challenging. Several participants described making no attempt to link without a card or referral letter, anticipating they would not be believed by staff if they presented without the necessary paperwork.

*No, I have not been [to the clinic], let me not lie… because I do not have a referral letter… because they cannot believe me if I went and showed up at the hospital saying that I was in prison, they would not believe me, they will think that I am lying.* —Male, aged 33 years (status deteriorated)

Navigating community health systems was not always straightforward and often hampered by highly bureaucratic requirements at the receiving clinic in the community.

Several participants who did not have the correct documents (either lost or never received) but still attempted to link to care described being dismissed and told to return to their previous clinic. This rejection by health care staff resulted in some participants choosing not to return to care at all. A female participant described her experience after misplacing her referral letter.

*The lady there [at the clinic] was very rude… the way she spoke to me… I felt like I had committed an offense again… From that time, I haven’t gone back again… I just left it. —*Female, aged 35 years (status deteriorated)

Some participants described feeling sensitive about being perceived as “bad people” by health care providers with implications for their willingness and ability to access care. Both men and women appeared more sensitive to their reception by health providers if they had never accessed ART in the community before. For these participants, the nature of their first experience with the health system was critical, with a positive first experience generally resulting in ongoing attendance at the clinic. This was exemplified by a participant who, despite initial anxiety about not having their paperwork in order, remained committed to attending their clinic visits after a highly positive first encounter.

*They received me properly. They were not upset with me. They happily received me… And they welcomed me and encouraged me… I come for every appointment they give me. —*Male, aged 44 years (unchanged favorable status)

### Enabling Characteristics: From Strong Formal and Informal Support Systems to Family-Only Support

#### Pre-Release: Informal Peer Supports

Pre-release, male and female participants both described grueling work requirements, poor sanitation and nutrition, overcrowding, and untenable sleeping arrangements in their correctional facility. These excessively harsh conditions, coupled with anxiety about the well-being of loved ones on the outside, led participants to describe high levels of anxiety, a sense of hopelessness, both hypersomnia and insomnia, suicidal thoughts, actual suicides, and “thinking too much.”

*In here we sleep like this [gestures sleeping while sitting upright], like the way I am showing you. One puts the head this side, one puts the head the other side, the other has his feet on somebody’s mouth, so that is tough …. You fail to think properly.* —Male, aged 42 years (unchanged favorable status)

Many participants recognized the negative impact that such mental stressors and mental health symptoms could have on ART adherence. They described the brain not working properly, being overwhelmed by their thoughts, or just giving up. Consequently, they sometimes felt incapable of making basic decisions in their daily life, including taking their medications.

Despite the highly stressful environment and participants’ recognition of the impact it had on medication adherence, most seemed unwilling to seek referrals to the clinic or support from peer educators for mental health issues. Concerns around privacy and the belief that mental health was not something that could be resolved with clinical care were central to explanations of this behavior.

*It [mental health] doesn’t concern them [health care workers].* —Participant, cell captain

*My problems? No, I can’t tell them [to peer educators] … Because if I tell them, people like to talk [and] talk and they can start laughing at me…* —Participant

Despite the difficult conditions in corrections, participants also described strong informal support within the prison community that simultaneously helped them to remain adherent to ART and manage mental health stressors. First, while many participants noted concerns about trusting other incarcerated persons or corrections officers, a number also spoke of a select group of incarcerated PLHIV who provided them with informal psychosocial support (both for HIV and mental health issues) and ART adherence counseling. In addition, in the absence of family support, both men and women described these informal networks as critical to ensuring adequate access to food.

*In here, people don’t starve, they don’t leave you to starve, even when you don’t have food, they will make an effort to give you something.* —Female, aged 33 years (became LTFU)

Some participants spoke of a select group of incarcerated PLHIV who provided them with informal psychosocial support and ART adherence counseling.

Second, male participants, in particular, described how concerns around an individual’s mental health could be escalated to cell captains, who would then consult with other cell captains, religious figures (typically pastors), or, in serious cases, sympathetic duty officers, who may be able to provide logistical support, such as facilitating contact with family or advocating for a transfer to a facility closer to home.

*Firstly, if you have any problem or any thoughts in prison we go to the captains in our cells. Then if he fails to solve the problem, he will go to the other captains and explain to them that this person has these thoughts or he has this problem. —*Male, aged 32 years (status deteriorated)

However, there were no descriptions of a similar system of escalating concerns through cell captains and beyond within the female section, where concerns around privacy featured more prominently in participant responses.

*Where do you even start from? You don’t open up to anyone… you keep it to yourself… If they hear you, even the people passing, because inside here, even the inmates are against each other. If they hear you telling the peer educator they will go and report.* —Female, aged 33 years (unchanged favorable status)

#### Pre-Release: Normalization of HIV

Many participants also associated their treatment adherence during incarceration with the non-stigmatizing environment of prisons with respect to HIV. This manifested in a number of ways. First, many participants who had either been diagnosed with HIV while incarcerated or who were previously not on ART noted the considerable time spent by health care providers in delivering counseling and care, including dedicated follow-up if incarcerated persons did not initiate ART on their first clinic visit. This care and follow-up were instrumental in helping incarcerated individuals internalize and accept their HIV diagnosis in a way that they had not experienced previously in the community.

*At first I didn’t understand. Until I think help came from the counselors. They are the ones that made me understand [pause] that I am positive … they kept finding me and talking to me … it felt like we talked for hours… they made me [feel that it is OK] … so it is just this thing … it is OK.* —Male, aged 34 years (status deteriorated)

Second, participants noted that the high HIV prevalence within the incarcerated population had a destigmatizing effect because living with the disease was largely normalized. Third, some male incarcerated persons noted that the absence of women helped them feel less embarrassed about their HIV status. A participant explained that when he was “outside,” he felt a large amount of anxiety related to how his HIV status impacted his ability to form sexual relationships with women. Since the ability to have such relationships was virtually impossible in prison, a major source of anxiety relating to his HIV status had been temporarily removed.

*No, there is nothing because in prison there are only men; there is no other way like maybe you are scared that they [women] will know.* —Male, aged 41 years (unchanged favorable status)

Finally, participants described how prison rules made it a punishable offense to tease, attack, or otherwise stigmatize an incarcerated person living with HIV, helping to promote a culture in which externalized stigma was unacceptable. Punishments included being placed on the penal block (a segregated space) or having “notes” placed against their docket number, which could impact the likelihood of early release for good behavior.

*If you laugh at your friend or talk about their [HIV] status … it is a big offense in prison. They can even change you from your cell. They will discipline you for talking about your friend’s health.* —Male, aged 29 years (unchanged, undetectable, or suppressed)

#### Post-Release: No Support Other Than Family

After release, there was very little mention of any maintenance of the enabling relationships and informal structures that had acted as a critical support mechanism during incarceration. In fact, several participants described the desire to distance themselves from any associations they had made in corrections, often leading to a greater sense of isolation and lack of support.

Consequently, the only support potentially available to participants was from family members. Men and women who were living with supportive family members were generally engaged in HIV care and reported feeling motivated to stay healthy. These participants described family members as providing critical logistical support to attend clinical visits and giving reminders to take medication, as well as offering intensive psychosocial support.

*If I have a lot to think about … Usually my best friend is my wife. Yes, I don’t have many friends, … But my wife … the one who knows me much, much better is my wife. She knows when I am happy, she knows when I am not happy. So, when I am very quiet then she will be concerned, “Why are you very quiet today? What are you thinking about?”* —Male, aged 50 years (status improved)

Participants described how a supportive family member could impact all elements of their lives, including beyond HIV care, particularly through financial support and housing, in ways that contributed to their overall health and ability to avoid reincarceration.

*…It depends on the family where you are going to be staying, if your family loves you … If they don’t love you that much, then this is where you find someone going back to jail after 2 or 3 days.* —Male, aged 29 years (unchanged favorable status)

Participants described how a supportive family member could impact their lives in ways that contributed to their overall health and ability to avoid reincarceration.

Such support was particularly important for participants suffering from alcohol or substance use or other mental health disorders. For example, a male participant, who described himself as suffering from chronic alcoholism, noted the role his wife (who was also living with HIV) played in ensuring his daily adherence to ART by collecting his medication and giving him daily reminders.

*My wife reminds me … If I forget, she tells me you are supposed to go on this day to the hospital that is how she reminds me.* —Male, aged 43 years (unchanged favorable status)

The quote above also highlights a critical distinction between male and female study participants when describing family support. In the case of male participants, regardless of their marital status, support was almost always provided by female family members, such as mothers, wives, and sisters. Female participants—who were nearly all single, separated, divorced, or widowed—more frequently commented on a lack of support from their families and noted facing additional hardships compared to their married peers. This, in turn, created additional stress and anxiety.

*The way I see it, it can be difficult outside. Why I see it that way is maybe you might not have transport at home and you are sick … like for us who are on medication, when you take the medication and then you feel bad, it is not everyone with a husband, others don’t have husbands …* —Female, aged 46 years (unchanged favorable status)

This isolation from family may be a critical factor in derailing effective linkage to care. Nearly every participant who reported a fractured family relationship was not engaged in HIV care at the time of study follow-up.

### Need Characteristic: No Discharge Planning Leads to Big Shock

#### Pre-Release: Perception That All Will Be Well When Released

When asked to consider how their needs (such as being able to link to HIV care) would be met post-release, participants generally expressed high levels of confidence. This optimistic conceptualization of life after release extended to all facets of life. For example, many participants, both men and women, believed they would no longer be depressed, their family would welcome them, and they would never return to prison. While also generally optimistic, incarcerated people who had first initiated ART while in prison struggled to conceptualize what HIV care in the community would look like. When asked to conceptualize how they would meet basic subsistence needs, such as housing, money, and food, many participants expressed a perception that “all will be well.” Many participants, particularly those with long incarceration periods, were unable to articulate exactly how these needs would be met.

#### Post-Release: Realization That All Is Not Well

Contrary to the optimistic accounts in participants’ pre-release interviews, many participants described a highly stressful transition back into the community as they struggled to meet basic subsistence needs associated with housing, finances, and food. This was driven by a combination of financial worries and stigma (both internalized and external) relating to their status as formerly incarcerated persons. Many men and women had lost their jobs during incarceration, and the stigmatization, both perceived and actual, of individuals with an incarceration history made seeking employment after release fraught and difficult. The inability to secure employment compounded participants’ dependence on others—often family—and many described the stress and shame that this dependence invoked.

*If you call a friend, sometimes they will just say, “hello, how are you?” You see … But when I came out of prison you call the same friend, this time they delay answering the phone. It’s like they know you would ask for help. You know that affects your mind you could say. This is the same guy we used to chat and he couldn’t delay answering my phone, but this time it will take time, there will be some quiet and even the tone you can read between the lines to say, “OK I don’t think this guy is thinking good about me” … that thing [shame] still affects you …* —Male, aged 50 years (status improved)

Contrary to the optimistic pre-release accounts, many participants described a highly stressful transition back into the community, driven by both financial worries and internalized and external stigma.

Many participants also felt that their incarceration experience “branded” them as dishonest persons, indicating a degree of internalized stigma. This impacted many aspects of their lives, including relationships, employment opportunities, and interactions with the health system, as well as their own sense of self-worth. For example, the same male participant felt his identity as a father, husband, and provider had been destroyed.

*After being released, the stigma I had was [from] being in prison, being an ex-convict. You know people look at you as being a thief in whatever way … That is the thing because people would ask you, “When did you come out from jail?” … You know, being a family man … head of the house … I am supposed to supply my family. But at the moment I am not doing that … Me, I am sensitive to some of these things.* —Male, aged 50 years (status improved)

Many participants whose viral load status deteriorated described resorting to either drinking alcohol and/or smoking marijuana as a mechanism to cope with their extreme sense of isolation or boredom. Others shared that while they had faced ostracization from many of their peers, it was often only their friends who also engaged in substance use who didn’t reject them post-release.

*[I drink] almost every day … Almost every day, but it is not that kind of drinking where you sit, you can’t find me in the morning normal. I always take alcohol. I drank yesterday and I will drink again […] You just have fun because we are used, we are drunks so we drink to be happy and be with friends. These friends they still talk to me. They are the only ones. So, I am happy when I am with them.* —Male, aged 41 years (unchanged unfavorable status)

The consequences of reported excessive substance use included being further impoverished, isolated from family, and unable to keep medical appointments or take medication.

## DISCUSSION

This is the first study from SSA, to our knowledge, that qualitatively describes the experiences and perspectives of incarcerated PLHIV as they transition from corrections into the community. Our study draws upon the lived experiences of 27 participants who recounted their journey to HIV care over the period spanning incarceration and release, plus 23 additional pre-release accounts, and was nested within a larger cohort study that linked those accounts to clinical and behavioral information. Our findings suggest that the highly structured and prescriptive nature of the corrections health system, while paradoxically contributing to adherence during incarceration, does not strengthen or build the self-efficacy required for incarcerated people to navigate an often indifferent, unaccommodating, and inflexible community health system upon release. This, coupled with inadequate counseling and discharge planning before release, as well as no case management or psychosocial support after release, can shock releasees when facing stark post-release realities of financial stress, unmet basic needs, social isolation, and stigma associated with incarceration, all of which contribute to poor mental health, including substance use issues. While our findings do echo results previously reported from North America, we describe stressors that are heightened as a result of more severe resource limitations, and enabling and need characteristics that are relatively unique to countries with under-resourced health and correctional systems. These factors contribute to post-release trajectories for incarcerated PLHIV in Zambia that differ in several ways from those in a high-income setting like the United States.

First, while financial and housing vulnerability has been well documented as a risk factor for poor linkage to HIV care post-release,^24^^–^^26^^,^^34^^,^^35^ the frequency with which these scenarios were recounted by our study participants was striking. The excessive vulnerability to extreme poverty experienced by newly released justice-involved persons in sub-Saharan African countries is well documented^36^^–^^38^; however, its relationship to HIV linkage post-release remains underexplored, as are support mechanisms to mitigate these vulnerabilities. Our findings suggest that any post-release HIV transitional care program in Zambia must incorporate access to and support for basic subsistence needs before other need characteristics can be addressed.

Our findings suggest that any post-release HIV transitional care program in Zambia must first address access to, and support for, basic subsistence needs.

Second, while the role of a supportive family has been noted as an important enabling factor in linkage to care in studies from the United States,^27^^,^^39^^,^^40^ within this cohort, there appeared to be no other community support mechanisms in place (or at least none known or obvious to participants). Although this echoes the importance of family in providing support for PLHIV generally, as reported in other countries in SSA, due to the complete lack of other support systems,^41^ this also highlights how justice-involved people who have fallen out with family—an unavoidable reality for many—have few or no other social safety net options and are at heightened risk of HIV care interruption. While certainly not an exclusive driver of HIV care engagement, the importance of family in the Zambian setting is illustrated by the fact that nearly every participant in our study who reported a fractured family relationship was not in HIV care at study follow-up.

Finally, despite the small sample of women in our study, our findings provide further evidence of the unique challenges faced by female justice-involved PLHIV in the Zambian setting.^42^ Again, justice-involved women have been previously identified as being at higher risk of falling out of care post-release compared to men^43^^,^^44^ as well as being especially vulnerable due to engaging in transactional or paid sex work, experiencing more domestic and intimate partner violence, and being more likely to report histories of sexual abuse.^45^ However, beyond these risk factors, the women in this cohort appeared to experience stressors exacerbated by the gender norms and structures of a highly patriarchal society.^46^ In particular, women at release more frequently reported social isolation, psychological distress, and family abandonment compared to male participants. Family abandonment made them particularly vulnerable to falling out of HIV care, given the complete lack of alternative support mechanisms, as described above. While services are also lacking for men, future work should also consider the unique needs of female justice-involved PLHIV, especially in light of increasing rates of female incarceration in Africa and globally.^47^

In addition to the relatively unique results described above, we also noted several findings that have been reported previously in both SSA and North America. First, similar to a handful of prior studies,^48^^–^^50^ participants in our study described a supportive environment for initiating and sustaining ART during incarceration, highlighting the formal and informal support structures (e.g., structured ART delivery system, informal support networks, and the normalization of HIV) within at least some of Zambia’s prisons. While many participants reported being able to navigate and link to care upon release, the stories of those who did not highlight the significant structural and socioeconomic challenges faced by a highly vulnerable population living in a state of flux. These challenges included facing inflexible and bureaucratic linkage procedures at community clinics. Successful linkage to community care was strongly influenced by the possession of a single printed (i.e., hard copy) letter provided by the correctional facility at release. Not receiving or misplacing this letter proved to be a substantial barrier, which was especially difficult to surmount for releasees who were socially isolated, financially stressed, or facing mental health or substance use issues. Additional work is needed to (1) train health workers in the national HIV program about issues faced by justice-involved PLHIV and (2) strengthen person-centered care by simplifying processes and providing more support.

More work is needed to train health workers on issues facing justice-involved PLHIV and to strengthen person-centered care.

Second, our findings highlighted the central importance of the first post-release encounter with the community health system for setting a positive trajectory for HIV care continuity. While negative first encounters with health care staff have been identified as a critical reason for failing to link to HIV care in the general population,^51^^–^^53^ this challenge may be even more significant for PLHIV newly released from prison who experience heightened anxiety and compounding internalized stigma relating to their HIV status, incarceration history, and social standing.

Third, similar to findings from the United States, our study suggests a potential role for pre-release discharge planning, case management, and peer-navigation support systems to assess releasees’ subsistence, psychosocial, and medical needs, as well as to help justice-involved PLHIV develop problem-solving skills and understand their HIV care and medical history.^25^^,^^30^^,^^34^^,^^54^^–^^60^ While acceptability studies evaluating differentiated care delivery models for justice-involved PLHIV in South Africa^61^ show promise, how such approaches could be adapted for and implemented within the Zambian correctional context requires further exploration.

### Limitations

Our study has several limitations. First, our study sample may have included participants more likely to be engaged in HIV care. Our participants were sampled from incarcerated persons known to be living with HIV and who had successfully engaged in HIV care in the correctional facility clinic. Participants often mentioned “another group” of incarcerated PLHIV who, for a variety of reasons, deliberately avoided clinical care and treatment. The size and significance of this group is unclear, and the perspectives of these potentially harder-to-reach incarcerated persons are not included here. Similarly, post-release interviews were conducted with participants who study staff were able to contact and who were willing to engage with the study. Second, while familiarity between study staff and some participants provided a degree of rapport, it may also have encouraged social desirability bias among participants who wanted to appear as doing well during interviews. Taken together, these factors suggest that the challenges faced by releasees may be even more severe or numerous than those presented here. Third, for operational feasibility reasons, study participation was limited to releasees who were planning to live in Lusaka and, as such, may not be generalizable to all justice-involved PLHIV, particularly those who may be highly mobile or who return to rural settings. Finally, the rapid and often unpredictable nature of release scheduling meant that enrollment of “remandees” awaiting trial was challenging, resulting in nearly all participants of this study being sentenced persons. Thus, our results may not be generalizable to the estimated one-quarter of incarcerated persons in Lusaka who are remandees. These individuals are highly underserved and face their own distinctive challenges at release, which may require uniquely tailored interventions and support.

## CONCLUSION

Our findings suggest that health gains made during incarceration may be lost for justice-involved PLHIV after prison release. These losses may be felt most acutely by releasees who are female, estranged from family, have no prior experience accessing HIV services in the community, and/or experience comorbid mental health or substance use conditions. Releasees are expected to navigate an inflexible and intimidating health care system in the community without support, alongside myriad concomitant financial, social, and psychological stressors, such as poverty, gender inequality, and mental health issues. Releasees with poor self-efficacy and problem-solving skills may require coaching, case management, and/or peer navigation to engage effectively with the community health care system. The first encounter with community health services after release appears critical and may set a trajectory toward poor or sustained care engagement, depending on the quality of the encounter. Training for health care providers should address the needs of recently released justice-involved PLHIV to better provide client-centered services. Additional services, including psychosocial, socioeconomic, and gender-responsive supports, may be helpful in overcoming the multilevel barriers to care identified in this study.

## Supplementary Material

22-00444-Topp-Supplement.pdf

## Notes

**Peer Reviewed**

**First published online:** April 19, 2023.

## References

[1] Walmsley R. *World Prison Population List (tenth edition)*. International Centre for Prison Studies; 2013. Accessed March 21, 2023. https://www.prisonstudies.org/sites/default/files/resources/downloads/wppl_10.pdf

[2] Zambia. World Prison Brief. Accessed March 21, 2023. https://www.prisonstudies.org/country/zambia

[3] Henostroza G, Topp SM, Hatwiinda S, et al. The high burden of tuberculosis (TB) and human immunodeficiency virus (HIV) in a large Zambian prison: a public health alert. PLoS One. 2013;8(8):e67338. 10.1371/journal.pone.0067338. 23967048 PMC3743881

[4] Maggard KR, Hatwiinda S, Harris JB, et al. Screening for tuberculosis and testing for human immunodeficiency virus in Zambian prisons. Bull World Health Organ. 2015;93(2):93–101. 10.2471/BLT.14.135285. 25883402 PMC4339958

[5] Simooya OO, Sanjobo NE, Kaetano L, et al. ‘Behind walls’: a study of HIV risk behaviours and seroprevalence in prisons in Zambia. AIDS. 2001;15(13):1741–1744. 10.1097/00002030-200109070-00023. 11546955

[6] Telisinghe L, Charalambous S, Topp SM, et al. HIV and tuberculosis in prisons in sub-Saharan Africa. Lancet. 2016;388(10050):1215–1227. 10.1016/S0140-6736(16)30578-5. 27427448 PMC6182190

[7] Kagujje M, Somwe P, Hatwiinda S, et al. Cross-sectional assessment of tuberculosis and HIV prevalence in 13 correctional facilities in Zambia. BMJ Open. 2021;11(9):e052221. 10.1136/bmjopen-2021-052221. 34580101 PMC8477336

[8] Republic of Zambia. Ministry of Health (MOH). *Zambia Population-Based HIV Impact Assessment (ZAMPHIA) 2016*. MOH; 2017. Accessed March 21, 2023. https://stacks.cdc.gov/view/cdc/50016

[9] Joint United Nations Programme on HIV/AIDS (UNAIDS). *Understanding Fast-Track Accelerating Action to End the AIDS Epidemic by 2030*. UNAIDS; 2015. Accessed March 21, 2023. https://www.unaids.org/sites/default/files/media_asset/201506_JC2743_Understanding_FastTrack_en.pdf

[10] World Health Organization (WHO). *Consolidated Guidelines on HIV Prevention, Diagnosis, Treatment and Care for Key Populations*. WHO; 2016. Accessed March 21, 2023. https://www.who.int/publications-detail-redirect/9789241511124 25996019

[11] Davies NECG, Karstaedt AS. Antiretroviral outcomes in South African prisoners: a retrospective cohort analysis. PLoS One. 2012;7(3):e33309. 10.1371/journal.pone.0033309. 22470448 PMC3310000

[12] Herce ME, Hoffmann CJ, Fielding K, et al. Universal test-and-treat in Zambian and South African correctional facilities: a multisite prospective cohort study. Lancet HIV. 2020;7(12):e807–e816. 10.1016/S2352-3018(20)30188-0. 32763152

[13] Human Rights Watch. *Unjust and Unhealthy: HIV, TB, and Abuse in Zambian Prisons*. Human Rights Watch; 2010. Accessed March 21, 2023. https://www.hrw.org/sites/default/files/reports/zambia0410webwcover.pdf

[14] Mpawa H, Kwekwesa A, Amberbir A, et al. Virological outcomes of antiretroviral therapy in Zomba central prison, Malawi; a cross‐sectional study. J Int AIDS Soc. 2017;20(1):21623. 10.7448/IAS.20.1.21623. 28782332 PMC5577730

[15] Southern African Development Community (SAD). *Minimum Standards for HIV and AIDS, TB, Hepatitis B and C, and Sexually Transmitted Infections Prevention, Treatment, Care and Support in Prisons in the SADC Region*. SADC; 2009. Accessed March 21, 2023. https://www.sadc.int/fr/file/2900/download?token=ffmTB2Ro

[16] Joint United Nations Programme on HIV/AIDS (UNAIDS). *The Gap Report*. UNAIDS; 2014. Accessed March 21, 2023. https://www.unaids.org/sites/default/files/media_asset/UNAIDS_Gap_report_en.pdf 12349391

[17] Wettstein C, Mugglin C, Egger M, et al; IeDEA Southern Africa Collaboration. Missed opportunities to prevent mother-to-child-transmission. AIDS. 2012;26(18):2361–2373. 10.1097/QAD.0b013e328359ab0c. 22948267 PMC3741537

[18] Meyer JP, Cepeda J, Wu J, Trestman RL, Altice FL, Springer SA. Optimization of human immunodeficiency virus treatment during incarceration: viral suppression at the prison gate. JAMA Intern Med. 2014;174(5):721–729. 10.1001/jamainternmed.2014.601. 24687044 PMC4074594

[19] Spaulding A, Stephenson B, Macalino G, Ruby W, Clarke JG, Flanigan TP. Human immunodeficiency virus in correctional facilities: a review. Clin Infect Dis. 2002;35(3):305–312. 10.1086/341418. 12115097

[20] Mabuto T, Woznica DM, Lekubu G, et al. Observational study of continuity of HIV care following release from correctional facilities in South Africa. BMC Public Health. 2020;20(1):324. 10.1186/s12889-020-8417-2. 32164628 PMC7068979

[21] Tran NT, Baggio S, Dawson A, et al. Words matter: a call for humanizing and respectful language to describe people who experience incarceration. BMC Int Health Hum Rights. 2018;18(1):41. 10.1186/s12914-018-0180-4. 30445949 PMC6240232

[22] Harris JB, Hatwiinda SM, Randels KM, et al. Early lessons from the integration of tuberculosis and HIV services in primary care centers in Lusaka, Zambia. Int J Tuberc Lung Dis. 2008;12(7):773–779. 18544203

[23] Topp SM, Moonga CN, Luo N, et al. Mapping the Zambian prison health system: an analysis of key structural determinants. Glob Public Health. 2017;12(7):858–875. 10.1080/17441692.2016.1202298. 27388512

[24] Dennis AC, Barrington C, Hino S, Gould M, Wohl D, Golin CE. “You’re in a world of chaos”: experiences accessing HIV care and adhering to medications after incarceration. J Assoc Nurses AIDS Care. 2015;26(5):542–555. 10.1016/j.jana.2015.06.001. 26188413 PMC4540691

[25] Sidibe T, Golin C, Turner K, et al. Provider perspectives regarding the health care needs of a key population: HIV-infected prisoners after incarceration. J Assoc Nurses AIDS Care. 2015;26(5):556–569. 10.1016/j.jana.2015.05.001. 26279385 PMC4542020

[26] Ghose T, Ali S, Shubert V, Stanton M, Walker L, Chaudhuri S. “It’s my room and my life”: housing’s influence on medication adherence for HIV-positive women released from incarceration. J Health Care Poor Underserved. 2019;30(1):182–201. 10.1353/hpu.2019.0015. 30827977

[27] Sun S, Crooks N, Kemnitz R, Westergaard RP. Re-entry experiences of Black men living with HIV/AIDS after release from prison: intersectionality and implications for care. Soc Sci Med. 2018;211:78–86. 10.1016/j.socscimed.2018.06.003. 29913303 PMC6364300

[28] Goodman-Meza D, Shoptaw S, Weiss RE, et al. Methamphetamine use drives decreases in viral suppression for people living with HIV released from a large municipal jail: results of the LINK LA clinical trial. Drug Alcohol Depend. 2019;202:178–184. 10.1016/j.drugalcdep.2019.05.007. 31352308 PMC6686887

[29] Haley DF, Golin CE, Farel CE, et al. Multilevel challenges to engagement in HIV care after prison release: a theory-informed qualitative study comparing prisoners’ perspectives before and after community reentry. BMC Public Health. 2014;14(1):1253. 10.1186/1471-2458-14-1253. 25491946 PMC4295310

[30] Khawcharoenporn T, Zawitz C, Young JD, Kessler HA. Continuity of care in a cohort of HIV-infected former jail detainees. J Correct Health Care. 2013;19(1):36–42. 10.1177/1078345812458246. 23023657

[31] Gelberg L, Andersen RM, Leake BD. The Behavioral Model for Vulnerable Populations: application to medical care use and outcomes for homeless people. Health Serv Res. 2000;34(6):1273–1302. 10654830 PMC1089079

[32] Saunders JB, Aasland OG, Babor TF, De La Fuente JR, Grant M. Development of the alcohol use disorders identification test (AUDIT): WHO collaborative project on early detection of persons with harmful alcohol consumption‐II. Addiction. 1993;88(6):791–804. 10.1111/j.1360-0443.1993.tb02093.x. 8329970

[33] Berman AH, Bergman H, Palmstierna T, Schlyter F. Evaluation of the Drug Use Disorders Identification Test (DUDIT) in criminal justice and detoxification settings and in a Swedish population sample. Eur Addict Res. 2005;11(1):22–31. 10.1159/000081413. 15608468

[34] Booker CA, Flygare CT, Solomon L, et al; EnhanceLink Study Group. Linkage to HIV care for jail detainees: findings from detention to the first 30 days after release. AIDS Behav. 2013;17(Suppl 2):128–136. 10.1007/s10461-012-0354-3. 23224290

[35] Hu C, Jurgutis J, Edwards D, et al. “When you first walk out the gates…where do [you] go?”: barriers and opportunities to achieving continuity of health care at the time of release from a provincial jail in Ontario. PLoS One. 2020;15(4):e0231211. 10.1371/journal.pone.0231211. 32275680 PMC7147766

[36] Jefferson AM. Traversing sites of confinement. Theor Criminol. 2010;14(4):387–406. 10.1177/1362480610370832

[37] Ngozwana N. Adult offenders’ perceptions of rehabilitation programs in Africa. Aust J Adult Learn. 2017;57(2):217–241.

[38] Osayi KK. Socio-cultural factors affecting reintegration of discharged prisoners in Anambra State, South East, Nigeria. Mediterr J Soc Sci. 2013;4(10):775. 10.5901/mjss.2013.v4n10p775

[39] Rozanova J, Brown SE, Bhushan A, Marcus R, Altice FL. Effect of social relationships on antiretroviral medication adherence for people living with HIV and substance use disorders and transitioning from prison. Health Justice. 2015;3(1):18. 10.1186/s40352-015-0030-6. 26709367 PMC4684583

[40] Rabinovich R, Owczarzak J, Mabuto T, Ntombela N, Woznica D, Hoffmann CJ. Social support needs of HIV-positive individuals reentering community settings from correctional facilities in Johannesburg, South Africa. AIDS Care. 2022;34(10):1347–1354. 10.1080/09540121.2021.1990200. 34668791 PMC9018888

[41] Mokomane Z. Social protection as a mechanism for family protection in sub‐Saharan Africa. Int J Soc Welf. 2013;22(3):248–259. 10.1111/j.1468-2397.2012.00893.x

[42] Topp SM, Moonga CN, Mudenda C, et al. Health and healthcare access among Zambia’s female prisoners: a health systems analysis. Int J Equity Health. 2016;15(1):157. 10.1186/s12939-016-0449-y. 27671534 PMC5037633

[43] Erickson M, Shannon K, Sernick A, et al. Women, incarceration and HIV. AIDS. 2019;33(1):101–111. 10.1097/QAD.0000000000002036. 30289811 PMC6350912

[44] Beckwith C, Castonguay BU, Trezza C, et al. Gender differences in HIV care among criminal justice-involved persons: baseline data from the CARE+ Corrections Study. PLoS One. 2017;12(1):e0169078. 10.1371/journal.pone.0169078. 28081178 PMC5231337

[45] United Nations Office on Drugs and Crime (UNODC). *Women and HIV in Prison Settings*. UNODC; 2008. Accessed March 21, 2023. https://www.unodc.org/documents/middleeastandnorthafrica/drug-prevention-health-publications/WOMEN_AND_HIV_IN_PRISON_SETTINGS.pdf

[46] Chileshe KK. Effects of a Patriarchal Society on a Zambian Woman’s Mindset: A Case Study of Lusaka Central. Undergraduate thesis. Rusangu University; date unknown.

[47] Penal Reform International (PRI). *Global Prison Trends 2021.* PRI; 2021. Accessed March 21, 2023. https://www.penalreform.org/global-prison-trends-2021

[48] Topp SM, Sharma A, Chileshe C, Magwende G, Henostroza G, Moonga CN. The health system accountability impact of prison health committees in Zambia. Int J Equity Health. 2018;17(1):74. 10.1186/s12939-018-0783-3. 30244684 PMC6151934

[49] Topp SM, Sharma A, Moonga CN, Chileshe C, Magwende G, Henostroza G. Evaluation of a health system strengthening initiative in the Zambian prison system. BMJ Glob Health. 2018;3(1):e000614. 10.1136/bmjgh-2017-000614. 29564162 PMC5859816

[50] Topp SM, Chetty-Makkan CM, Smith HJ, et al. “It’s not like taking chocolates”: factors influencing the feasibility and sustainability of universal test and treat in correctional health systems in Zambia and South Africa. Glob Health Sci Pract. 2019;7(2):189–202. 10.9745/GHSP-D-19-00051. 31249019 PMC6641809

[51] Sanga ES, Mukumbang FC, Mushi AK, Lerebo W, Zarowsky C. Understanding factors influencing linkage to HIV care in a rural setting, Mbeya, Tanzania: qualitative findings of a mixed methods study. BMC Public Health. 2019;19(1):383. 10.1186/s12889-019-6691-7. 30953503 PMC6451278

[52] Horter S, Thabede Z, Dlamini V, et al. “Life is so easy on ART, once you accept it”: Acceptance, denial and linkage to HIV care in Shiselweni, Swaziland. Soc Sci Med. 2017;176:52–59. 10.1016/j.socscimed.2017.01.006. 28129547

[53] Nyato D, Nnko S, Komba A, et al. Facilitators and barriers to linkage to HIV care and treatment among female sex workers in a community-based HIV prevention intervention in Tanzania: a qualitative study. PLoS One. 2019;14(11):e0219032. 10.1371/journal.pone.0219032. 31743336 PMC6863533

[54] Loeliger KB, Altice FL, Desai MM, Ciarleglio MM, Gallagher C, Meyer JP. Predictors of linkage to HIV care and viral suppression after release from jails and prisons: a retrospective cohort study. Lancet HIV. 2018;5(2):e96–e106. 10.1016/S2352-3018(17)30209-6. 29191440 PMC5807129

[55] Hammett TM, Donahue S, LeRoy L, et al. Transitions to care in the community for prison releasees with HIV: a qualitative study of facilitators and challenges in two states. J Urban Health. 2015;92(4):650–666. 10.1007/s11524-015-9968-x. 26022666 PMC4524841

[56] Westergaard RP, Hochstatter KR, Andrews PN, et al. Effect of patient navigation on transitions of HIV care after release from prison: a retrospective cohort study. AIDS Behav. 2019;23(9):2549–2557. 10.1007/s10461-019-02437-4. 30790170 PMC6703963

[57] Dauria EF, Kulkarni P, Clemenzi-Allen A, Brinkley-Rubinstein L, Beckwith CG. Interventions designed to improve HIV continuum of care outcomes for persons with HIV in contact with the carceral system in the USA. Curr HIV/AIDS Rep. 2022;19(4):281–291. 10.1007/s11904-022-00609-x. 35674879 PMC9175158

[58] Fuller SM, Koester KA, Maiorana A, et al. “I don’t have to do this all by myself”: systems navigation to ensure continuity of HIV care for persons leaving prison. AIDS Behav. 2019;23(Suppl 1):14–24. 10.1007/s10461-018-2050-4. 29442194

[59] Woznica DM, Fernando NB, Bonomo EJ, Owczarzak J, Zack B, Hoffmann CJ. Interventions to improve HIV care continuum outcomes among individuals released from prison or jail: systematic literature review. J Acquir Immune Defic Syndr. 2021;86(3):271–285. 10.1097/qai.0000000000002523. 33079904 PMC8495492

[60] Spaulding AC, Messina LC, Kim BI, et al. Planning for success predicts virus suppressed: results of a non-controlled, observational study of factors associated with viral suppression among HIV-positive persons following jail release. AIDS Behav. 2013;17(Suppl 2):203–211. 10.1007/s10461-012-0341-8. 23076719

[61] An Y, Ntombela N, Hoffmann CJ, Fashina T, Mabuto T, Owczarzak J. “That makes me feel human”: a qualitative evaluation of the acceptability of an HIV differentiated care intervention for formerly incarcerated people re-entering community settings in South Africa. BMC Health Serv Res. 2022;22(1):1092. 10.1186/s12913-022-08469-2. 36028825 PMC9415240

