# Supplementary material for: Experiences of Justice-Involved People Transitioning to HIV Care in the Community After Prison Release in Lusaka, Zambia: A Qualitative Study
Source: Glob Health Sci Pract. 2023 Apr 28;11(2):e2200444. doi: 10.9745/GHSP-D-22-00444 (PMC10141426; doi:10.9745/GHSP-D-22-00444)
Supplement: 22-00444-Topp-Supplement.pdf [file 22-00444-Topp-Supplement.pdf]

Supplement to: Smith HJ, Herce ME, Mwila C, et al. Experiences of justice-involved people transitioning to HIV care in the community after prison release in Lusaka, Zambia: a qualitative study. *Glob Health Sci Pract*. 2023;11(2):e2200444. https://doi.org/10.9745/GHSP-D-22-00444

SUPPLEMENT

1. Pre-Release Inmate In-Depth Interview Guide

Introductory Script (follows full verbal consenting process)

Please read: ["Hello. My name is ________________________. Thank you for agreeing to an interview today.

We are interested to hear about your experiences with health services and HIV treatment and care in prison/ corrections. We also want to know about your thoughts and feelings toward being an inmate / prisoner, having HIV, and getting support for your HIV in prisons / corrections. You do not have to answer any question that you do not want to.

I want to reassure you that the information you share is confidential. That is, what you say will not be shared with anyone outside the study or traced back to you in any way. While the information gathered during this interview will be combined with information from other interviews and shared with the Zambian Correctional Service and the Ministry of Health, no one will know who said it, when it was said, or where it was said. There are no 'right' or 'wrong' answers. Please feel free to ask me any questions if something is unclear.

Do you have any questions before we begin?"]

Part 1 - Background information:

This is to establish rapport…

a) To begin, please tell me a little about yourself and your family.
   [PROBES]
   - Age
   - Where are you from?
   - Marital status/ children

Part 2- Experiences with Prison/ Correctional Health Services

We are now going to talk about healthcare in the prison / corrections in general

a) What do you think about the health services here in prison/corrections?
   a. Are they effective / timely / efficient?
b) How have health workers at the clinic treated you?
c) How do you think that the healthcare you receive in prison/ corrections is different from the healthcare you have received on the outside?

Part 3 – HIV Care and Treatment Experiences

a) Are you receiving ART currently? If so, could you tell me a little bit about when you started receiving ART and what was it like for you starting ART? ***[If NOT receiving ART, SKIP to 2d]***
b) What's it been like to take ART/ ARVs while in prison/ corrections?
   - Please tell me about anyone who may have helped you take your ART/ARVs in prison/ corrections? How do they help?

- **PROBE**: Peer educators, cell captains, other inmates, HCWs, corrections officers

- Please tell me about anything that makes it hard to take ART/ ARVs while in prison/ corrections?

  PROBE: Stigma/ shame, availability of medicines/ ARVs, food, day labor/ "gangem", attitude of health workers/ corrections officers/ peer educators/ cell captains/ other inmates

c) If you were receiving ART before coming to prisons/ corrections, what was it like trying to stay on your ARVs / receive HIV care when you first entered the prison/ correctional facility?

[PROBES]

- Confirming HIV status
- ART interruption/ Loss or misplacement of ARVs
- Wait/ delays to see the clinician/ nurse

Part 4 - Future HIV Care and Other issues

a) How do you feel you will manage your HIV treatment in the long term after you leave prison/ corrections / are released to the community?
   a. **PROBE**: Where do you think you will see the doctor/receive care? Do you think you will have any problems doing this? Please describe.
   b. **PROBE**: How do you plan to get your medicine? Do you think you will have any problems doing this? Please describe.

b) Do you think you will tell people close to you that you were in prison/ corrections? That you are HIV-positive? Why or why not?

Part 5 – Predisposing and Enabling Factors / Health and Structural Issues

a) Can you tell me what are the major factors affecting your ability to stay healthy in prison/ corrections right now?
   - ***[PROBES]:*** Overcrowding / infrastructure; Hygiene / Clean water; Food/ Nutrition; Stigma/ Discrimination; Depression / Anxiety / Other Psychological Issues; Alcohol / Substance Use; Problems with other inmates and/or officers

b) Do you think these factors are the same or different for others? Please describe.
   - Why / why not?

c) If you are feeling sad or anxious, what do you do? Who do you talk to, if anyone, about these feelings?
   - Do you feel like you can talk about these problems with health workers at the clinic?
     - Please explain why or why not.
   - Do you feel like you can talk about these problems with peer health educators?
     - Please explain why or why not.
   - Do you feel like you can talk about these problems with other inmates?
     - Please explain why or why not.

d) Where would someone find alcohol in prison if they wanted to? Do you think this is a problem in the prison? Please explain.

e) Is there anything else you would like to share before we finish?

Please read: ["**Thank you very much for your time. That brings us to the end of the interview."]**

Supplement to: Smith HJ, Herce ME, Mwila C, et al. Experiences of justice-involved people transitioning to HIV care in the community after prison release in Lusaka, Zambia: a qualitative study. *Glob Health Sci Pract*. 2023;11(2):e2200444. https://doi.org/10.9745/GHSP-D-22-00444

2. Post-Release Inmate In-Depth Interview Guide

Introductory Script
Please read: ["Hello. My name is ___________________________. Thank you for agreeing to an interview today.

We are interested to hear about your experiences since leaving prison/ corrections, especially what it has been like getting HIV treatment and care and other health services in the community. We want to know your thoughts and feelings about dealing with HIV and getting support for your HIV in the community. We also would like to know about other behaviours and experiences you've had since returning to the community. You do not have to answer any question that you do not want to.

I want to assure you that the information you share is confidential. That is, what you say will not be shared with anyone outside the study or traced back to you in any way. While the information gathered during this interview will be combined with information from other interviews and shared with the Zambian Correctional Service and the Ministry of Health, no one will know who said it, when it was said, or where it was said. There are no 'right' or 'wrong' answers. Please feel free to ask me any questions if something is unclear.

Do you have any questions before we begin?"]

<u>Part 1 - Background information:</u>
This is to establish rapport…

b) To get started, I'm interested to learn about how things have been for you since your release from prison/corrections?

[PROBES]

- How long ago were you released from prisons/ corrections?
- Where are you staying / living currently (housing)?
- Who are you staying/ living with?
- How do you spend your time (work/ employment)?

<u>Part 2 - Experiences with Health Services in the Community</u>
Please read verbatim: ["We are now going to talk about your experiences with healthcare in the community."]

d) Tell me about how you get medical care now that you have left corrections/ prisons and live in the community?

[PROBES]

- Talk me through the steps you take from the time you decide whether or not to go to clinic until you've received care at the clinic?

e) What are some of the reasons why you may not go to the clinic when you need medical care?

<u>Part 3 – HIV Care and Treatment Experiences</u>

Supplement to: Smith HJ, Herce ME, Mwila C, et al. Experiences of justice-involved people transitioning to HIV care in the community after prison release in Lusaka, Zambia: a qualitative study. *Glob Health Sci Pract*. 2023;11(2):e2200444. https://doi.org/10.9745/GHSP-D-22-00444

d) Were you taking ARVs in prisons/corrections? ***[If NOT receiving ART in prison, SKIP to 3c]*** If you *were* taking ARVs/ ART in prisons/ corrections, what was it like trying to stay on your ARVs when you first left prison/ corrections? ***[THEN SKIP TO 3e]***
[PROBES]
- Referral from prison/ corrections / Figuring out where to go for HIV care
- ART interruption/ Loss or misplacement of ARVs
- Disclosure to family/ partner/ health workers
- Transportation
- Stigma
  - Ex-offender
  - HIV status

e) Issues at clinic (confirming status, waiting in cue, etc.)If you *were not* taking ARVs/ ART in prisons/ corrections, have you since started ART since leaving prisons/corrections? ***[IF YES, SKIP to 3d]***
- ***If NO – can you tell me why not? [THEN SKIP to 3f]***

[PROBES]
- Figuring out where to go for HIV care
- Disclosure to family/ partner/ health workers
- Stigma / Shame
  - Ex-offender
  - HIV status
  - Taking ARVs / going to ART clinic
- Competing needs/ demands (finding work, food, housing, etc.)

f) If you *did start* taking ARVs/ ART after release, what was it like starting ARVs after you left prison/ corrections?
[PROBES]
- Referral from prison/ corrections / Figuring out where to go for HIV care
- Transportation
- Disclosure to family/ partner/ health workers
- Stigma / Shame
  - Ex-offender
  - HIV status
  - Taking ARVs / going to ART clinic
- Support from others
- Issues at clinic (confirming status, waiting in cue, etc.)

g) Are you taking ART currently? ***[IF NO SKIP TO 3g]***
- If no, please tell me why you stopped.

[PROBES]
- Stigma / Shame
  - Ex-offender
  - HIV status
- Lack of support from others
- Accessing the clinic or medications/ refills
- Competing needs/ demands (finding work, food, housing, etc.)

h) What challenges do you face now, if any, with taking ARVs/ ART?
[PROBES]
- Stigma / Shame

    - Ex-offender
    - HIV status
    - Surrounding taking ARVs / going to ART clinic
- Support from others
- Remembering to take ARVs
- Accessing the clinic or medications/ refills
- Competing demands (finding work, food, housing, etc.)

g) Have you thought about ways to make sure you keep taking your ARVs over the long term? If so, what ways?

h) ***[ASK ALL WHO TOOK ARVs AT ANY POINT DURING INCARCERATION AND AFTER RELEASE]*** From your own experience, how has getting ARVs/ ART in the community been different from getting ARVs/ ART in prison/ corrections? How have these differences affected your health? Please explain.

[PROBES]

- Perceived timeliness / efficiency/ quality of ART/HIV services
- Availability of ARVs and other medicines
- Attitude of health workers / treatment by health workers
- Attitudes of people in the community versus inmates in prison/corrections
- Stigma
- Support to take ARVs/ ART (or go to clinic if NOT on ARVs/ ART)
  [PROBES]
    - Role of family/ friends
    - Role of peer educators
    - Role of health workers
    - Role of other social services/ government/ church groups/ NGOs

Part 4 – Predisposing and Enabling Factors / Health and Structural Issues

b) Can you tell me what are the major issues affecting your health right now?

[PROBES]

- Housing
- Food/ Nutrition
- Hygiene/ Clean water
- Healthcare Quality/ Availability
- Acceptance by family/ friends/ community
- Stigma/ Discrimination
- Depression / Anxiety / Thinking back to time in prison/ corrections
- Fear of police/ government/ re-incarceration
- Alcohol / Substance Use

c) Do you think these same issues affect other ex-offenders/ releasees too? Please explain why / why not?

d) If you are feeling sad or anxious, what do you do? Who do you talk to, if anyone, about these feelings?

- Do you feel like you can talk about these problems with health workers at the clinic?
    - Please explain why or why not.
- Do you feel like you can talk about these problems with peer educators at the clinic?
    - Please explain why or why not.
- Do you feel like you can talk about these problems with family and friends?
    - Please explain why or why not.

- Do you feel like you can talk about these problems with other ex-offenders/ people who have been in prison before?
  - Please explain why or why not.

Part 5 – Alcohol Use

a) Do you currently drink alcohol? ***[If NO, SKIP to Part 7]***

b) What alcoholic beverage(s) do you usually drink?

[PROBES]

- Beer (e.g. Mosi, Chibuku Shake Shake, etc.)
- Home-brewed drinks (e.g. Katata, etc.)
- Wine
- Spirits

c) Please tell me about the settings where you drink.

[PROBES]

- Sharing drinks/ Drinking alone
- Drinking venue
- People/ drinking "buddies" / social networks (e.g. friends, co-workers, ex-offenders)

d) What are some of the reasons that you drink alcohol?

[PROBES]

- To become inebriated / drunk
- Passing time/ boredom
- Socializing
- Sad/ Depressed/ Self-medication

Part 6 – Hazardous Alcohol Use

*For participants with AUDIT >8 or history of alcohol dependence…**[If NO, SKIP to Part 7]***

a) Have you thought about trying to stop or cut down on your drinking? If so, why? ***[If NO, SKIP to Part 7]***

[PROBES]

- Effects on Health
- Effects on Relationships
- Effects on Work / Livelihood

b) If you wanted to take action on this today, where do you think you could go or who would you talk to?

Part 7 – Drug Use

a) Do you currently use drugs? ***[If NO, SKIP to Part 8]***

b) Please tell me about the settings where you use drugs.

[PROBES]

- Sharing drugs/ Using drugs alone
- Drug use venue
- People/ Social networks (e.g. friends, co-workers, other ex-offenders)

c) What are some of the reasons that you use drugs?

[PROBES]

- To feel good/ get "high"
- Passing time/ boredom
- Socializing
- Sad/ Depressed/ Self-medication

Supplement to: Smith HJ, Herce ME, Mwila C, et al. Experiences of justice-involved people transitioning to HIV care in the community after prison release in Lusaka, Zambia: a qualitative study. *Glob Health Sci Pract*. 2023;11(2):e2200444. https://doi.org/10.9745/GHSP-D-22-00444

d) Have you thought about trying to stop or cut down on your drug use? If so, why? ***[If NO, SKIP to Part 8]***
[PROBES]
- Effects on Health
- Effects on Relationships
- Effects on Work / Livelihood

e) If you wanted to take action on this today, where do you think you could go or who would you talk to?

Part 8 - Other issues

c) Is there anything else you would like to share before we finish?
